# Supplementary material for: Patterns of Intron Gain and Loss in Fungi
Source: PLoS Biol. 2004 Nov 30;2(12):e422. doi: 10.1371/journal.pbio.0020422 (PMC532390; doi:10.1371/journal.pbio.0020422)
Supplement: Table S1 — Also available at http://genes.mit.edu/NielsenEtAl/. (4.3 MB ZIP). [file pbio.0020422.st001.zip › NielsenEtAl/html/976.html]

AN6640.1.NCU08886.1.MG09071.1.FG02121.1


```
 CLUSTAL W (1.82) Multiple Sequence Alignments - Introns Inserted


Sequence 1: NCU08886.1	407 aa
Sequence 2: MG09071.1	412 aa
Sequence 3: AN6640.1	419 aa
Sequence 4: FG02121.1	420 aa
Alignment Length: 430 aa
Number Identitical Residues: 119 aa
Alignment Score (without introns) 6473


MG09071.1 	-MRQSAESIVLGVVRDELREKDGDIR-GINDQ0PLY0IHAHPELAYEEFKAHDAFVSLLR
NCU08886.1	----MTDPTISTTIASAIQSNKSEIA-LIN--~-TH~IHSNPELAYLEFTAHSTLVHSLR
FG02121.1 	MPSATTISDVIGIARQTIASETKRLQSHLEKV~SLD~IHSHPELCYEEVFAHDTIFDFLE
AN6640.1  	MQLSASQLRYLKPVQIALRQYDEKLQ-DIN--~-HK0IWSNPELAYNEHQAHNNICELFE
          	     :           : .    :   ::       * ::***.* *  **. :   :.

MG09071.1 	KLQ---FEVTPHAYGVETAFSADYGSG----GRLVVYNAEYDALPGIGHACGHNLIASSS
NCU08886.1	RLGG--FTITSPAYGLPTSFEAEYGSG----GRLIIFNAEYDALPEIGHACGHNLIASAS
FG02121.1 	ANG---FTVTRHAYDISTCFEAEIGSG--EEEQTVIYCAEYDALPGIGHACGHNLIALSS
AN6640.1  	ALRSEGYSVRRHAYGLDTAFEIEYKHGDADNGRVLTFNAEYDALPGIGHACGHNLIATSS
          	   ...: :   **.: *.*. :   *.:.. : : : ******* *********** :*

MG09071.1 	LAAFLGLVAALKHHP--DQPGRVRLLGTPAEEGGGGKLKLIAAGAYKDAAASLMVHPGPS
NCU08886.1	LAAFLGVAAALKQS---GQPGRVRLLGTPAEEGGGGKLKLIEAGAYKGCDACLMVHPGPQ
FG02121.1 	ITAFVAIAKAMAETG-LAGKGKVRLLGTPAEEGGSGKVRLLDAGAFKGASAALMSHPTSM
AN6640.1  	IAAFIATCEAMKIAAPAGLVYTVRLLGTPAEESGGGKIRLLDSGAYKGVNACLMVHPAAL
          	::**:.   *:    .      **********.*.**::*: :**:*.  *.** ** . 

MG09071.1 	YLLAAPVEEVAFVR---MLANVKMRIYFSGRESHAAMAPWDGINALDAAMLSYNAVSMLR
NCU08886.1	SKLPPGINAVSYVR---MLANVKYRVYFTGREAHASIAPWHGVNALDAVCLSYNAVSMLR
FG02121.1 	GR-IKEVEGYAGAAGLVTIAASMISADFYGKSAHAGATPWQGHNALDAAVGSYVNISMLR
AN6640.1  	PPGQANLLSVAKPPEGGFLANDKVRVTFTGKPAHAAAAPWEGVNALDAVVAAYVNISLLR
          	      :   :       :*       * *: :**. :**.* *****.  :*  :*:**

MG09071.1 	QHIRPYERIHGVVRQGGERPNVTPSSTQVEYYIRSDTRAAAQKLWERVKLCFEGAAIATG
NCU08886.1	QQIRPEERIHGIFREAGTRPNVTPADCCVEYYVRSTTRRGAEELGKRVLKCFEGAALATG
FG02121.1 	QQIKPAERIHGVITDGGKAPNIIPDKTSIVYGVRSTTAAGCKDLLRKVKCCFDAAATATD
AN6640.1  	QQILPSQRVHGIIVNGGDRPNIIPWSASVDYYIRSPTLATLKPLTEKVVKCFEAAATATG
          	*:* * :*:**:. :.*  **: * .  : * :** *    : * .:*  **:.** **.

MG09071.1 	CGVRYEP~INSYADVRPSRALCREY-QAVQAPDTVSFSEPADFLAG--STDMG~DVCYEC
NCU08886.1	CEWRKED~LPAYFDVRPSKPLCKAWMEAVQPAGSVAWEDPTDFFQG--STDMG~NVCYEC
FG02121.1 	CTVKTKS2PLFYADQILNPVLCEAFTEEMRYLGDEFLPIAKETVSS--STDMG1NVSHVL
AN6640.1  	CKVEFQW2GPSYADVKSNEHICESYVSVMRAMGQHVLLNSPQGAGGSASTDMG1NVSYAI
          	*  . :     * *   .  :*. : . ::  .      . :   .::***** :*.:  

MG09071.1 	PGFHGAFGIDTEKGQSNHTPAFAAAAALDKSYKKAIECGRGMAMVGWRVLSDDAFAAEVQ
NCU08886.1	PGFHGVFGIETEDGAANHTKGFTKGAGTQDAFERALECGRAMAEVGWRVLADEEFAADVR
FG02121.1 	PSFHCSYGIHSGAAS-NHTPGFTAAAATPDALDRAIRVGRGLAMLGVYILHNPEFSTRMT
AN6640.1  	PGFHGIFFISAKSG--NHTPQFTDGAGSPNAYRRALDCAAGMAVTACYFLDDDDFARQVE
          	*.**  : * :  .  ***  *: .*.  .:  :*:  . .:*  .  .* :  *:  : 

MG09071.1 	KEWEEDMRIAKDT-
NCU08886.1	REWEVDMKVAGGN-
FG02121.1 	TAFKAAKAQADANI
AN6640.1  	EDFLRGGSL-----
          	  :
```
